# Subclinical primary aldosteronism and major adverse cardiovascular events: evidence for a continuum of renin-independent aldosterone excess and a proposal for early detection

**DOI:** 10.3389/fcvm.2026.1826678

**Published:** 2026-06-23

**Authors:** Atef Akoum, Mounir Hakim, Rola Kwayess, Bahaa El Deen Wehbeh, Lina Alaaeddine, Mohamed Nasser El Shabrawi, Ashesh Das, Akshay Kumar, Sreekant Avula, Abdallah Rebeiz, Jason Li

**Affiliations:** 1 Department of Internal Medicine, Hennepin Healthcare, Minneapolis, MN, United States; 2 Faculty of Medicine, American University of Beirut, Beirut, Lebanon; 3 School of Medicine, Division of Endocrinology and Metabolism, Indiana University, Indianapolis, IN, United States; 4 Department of Internal Medicine, American University of Beirut Medical Center, Beirut, Lebanon; 5 Division of Endocrinology and Metabolism, American University of Beirut Medical Center, Beirut, Lebanon; 6 Clinical Research Department, Aswan Heart Center, Magdi Yacoub Foundation, Aswan, Egypt; 7 Mayo Clinic, Rochester, MN, United States; 8 Department of Cardiothoracic Surgery, New York University Langone Health, New York, NY, United States; 9 Department of Endocrinology, University of Minnesota, Minneapolis, MN, United States; 10 Department of Cardiology, American University of Beirut Medical Center, Beirut, Lebanon; 11 Division of Cardiovascular Medicine, University of Massachusetts Chan Medical School, Worcester, MA, United States

**Keywords:** aldosterone, cardiovascular risk, hypertension, major adverse cardiovascular events, primary aldosteronism, renin suppression, subclinical primary aldosteronism

## Abstract

**Background:**

Primary aldosteronism (PA), a renin-independent state of aldosterone excess, is associated with excess risk of stroke, myocardial infarction, atrial fibrillation, heart failure, and chronic kidney disease compared with essential hypertension. Emerging evidence suggests that renin-independent aldosterone excess exists on a continuum rather than as a dichotomous disease state, including subclinical primary aldosteronism (sPA), even in normotensive individuals. Whether sPA is associated with major adverse cardiovascular events (MACE) prior to overt hypertension remains incompletely characterized.

**Objective:**

To synthesize available evidence linking renin suppression and mild aldosterone excess to cardiovascular outcomes.

**Methods:**

A narrative review of the literature was conducted using PubMed/MEDLINE, the Cochrane Library, and Web of Science from inception through May 2025, to identify cohort studies evaluating renin phenotype, aldosterone levels, and cardiovascular outcomes.

**Results:**

Suppressed renin and higher aldosterone levels, even within conventionally normal ranges, are associated with incident hypertension, left ventricular hypertrophy (LVH), atrial fibrillation, and cardiovascular events. In overt PA, significantly higher rates of cardiovascular diseases have been observed compared with blood pressure-matched essential hypertension. Emerging population-based data suggest a graded cardiovascular risk extending below traditional diagnostic thresholds.

**Conclusion:**

Biochemical phenotypes consistent with sPA may represent an early cardiometabolic state associated with elevated risk of MACE, even before overt PA criteria are met. The absence of standardized diagnostic thresholds and randomized interventional data currently limits clinical translation. Prospective trials evaluating renin-guided screening and early mineralocorticoid receptor antagonism are needed to determine whether intervention at the subclinical stage reduces long-term cardiovascular harm.

## 1 Introduction

Aldosterone, a key component of the renin–angiotensin–aldosterone system (RAAS), is instrumental in the management of blood pressure (BP) and water-mineral balance through mineralocorticoid receptors (MRs) expressed in epithelial tissues such as the distal nephron, colon, and sweat glands (1). However, MRs are also expressed in non-epithelial tissues, including cardiomyocytes, vascular smooth muscle cells, endothelial cells, and neurons, extending aldosterone's biological effects beyond sodium retention (2). Through widespread MR activation, excess aldosterone promotes oxidative stress, vascular inflammation (3, 4), and myocardial remodeling (5) at least partly independent of its hypertensive effects (1). Clinically, these processes have been associated with arterial stiffness, increased left ventricular mass, and ultimately major adverse cardiovascular events (MACE) (6–9).

Primary aldosteronism (PA), defined by autonomous, renin-independent aldosterone production, classically presents with resistant hypertension and in a minority of cases with hypokalemia (10, 11). Current screening strategies focus largely on these high-risk groups. Although overt PA is associated with greater cardiovascular morbidity than essential hypertension even after accounting for blood pressure levels, most studies do not stratify patients with PA by disease severity, leaving outcomes in milder forms of this syndrome poorly characterized (8).

Growing evidence supports a continuum of renin-independent aldosterone secretion extending into normotensive and mildly hypertensive individuals, a condition referred to as subclinical primary aldosteronism (sPA) (10, 12, 13). This concept is not entirely new, as early physiologic observations noted an inverse relationship between plasma renin activity and blood pressure, raising the probability that inappropriate MR activation might occur even in normotensive individuals (14, 15). Subsequent cohort studies formally identified autonomous, non-suppressible aldosterone secretion in normotensive populations, with prevalences of 13%–14% using standardized testing techniques (12, 13, 16, 17). Histopathologic analyses further revealed aldosterone-producing cell clusters (APCCs) harboring somatic mutations associated with overt PA are detectable in more than half of histologically normal adrenal glands in normotensive patients, providing a possible biological plausibility of early autonomous secretion (18, 19).

Despite guideline recommendations, PA screening remains substantially underutilized, with fewer than 2% of eligible patients evaluated in real-world practice (20–22). This raises concern that milder forms of renin-independent aldosteronism, including sPA, are frequently overlooked, potentially allowing aldosterone-mediated cardiovascular injury to accumulate before clinical recognition. Against this background, this review synthesizes emerging evidence linking sPA to cardiovascular risk, with the aim of proposing a hypothesis-generating framework that may inform the design of prospective screening and future interventional studies.

## 2 Methods

A narrative literature review was conducted to identify studies evaluating the relationship between subclinical primary aldosteronism and cardiovascular outcomes. Electronic databases including PubMed, MEDLINE, the Cochrane Library, and Web of Science were searched from inception till May 2025. Searches were limited to English-language publications.

The search strategy combined MeSH terms and free-text keywords using Boolean operators (AND, OR). Core terms included: “primary aldosteronism,” “subclinical primary aldosteronism,” “mild aldosterone excess,” “renin-independent aldosterone excess,” “suppressed renin,” “low-renin hypertension,” and “aldosterone-to-renin ratio.” These were combined with outcome terms including: “cardiovascular disease,” “major adverse cardiovascular events,” “MACE,” “incident hypertension,” “atrial fibrillation,” “left ventricular hypertrophy,” “arterial stiffness,” “heart failure,” “stroke,” and “cardiovascular mortality.” Similar strategies were applied across all three databases. Reference lists of all retrieved articles and key review papers were manually screened for additional eligible studies.

Studies were considered eligible for primary inclusion if they involved human subjects with a quantitative measure of aldosterone or renin suppression and reported at least one clinical or structural cardiovascular outcome. Studies restricted exclusively to overt primary aldosteronism defined by confirmed hypokalemia or resistant hypertension, without biochemical characterization of the subclinical spectrum, were excluded from the primary evidence base. Studies reporting only biochemical endpoints without cardiovascular outcomes were also excluded. Where relevant, mechanistic and therapeutic evidence from adjacent populations, including overt PA and heart failure cohorts, was incorporated in the treatment section as indirect supportive context, explicitly framed as extrapolative rather than directly applicable to sPA. Key data including study design, sample size, population characteristics, exposure definition, outcomes, follow-up, and degree of covariate adjustment were extracted and summarized in Tables 1, 2. Given the absence of a consensus on a diagnostic threshold for sPA, no single biochemical cutoff was applied as an inclusion criterion. The marked heterogeneity in exposure definitions across studies is documented in Table 2, which explicitly presents these as study-specific operational definitions rather than interchangeable diagnostic criteria. Throughout this review, the term sPA is used to denote a biochemical phenotype of renin-independent aldosterone excess falling below conventional thresholds for overt PA, as confirmed by the 2016 and 2025 Endocrine Society guidelines (11, 23). However, as a narrative rather than systematic review, this synthesis is not comprehensive and the literature selection process may be subject to selection bias, hence conclusions should be interpreted with caution.

**Table 1:** Major cohort studies evaluating the association between sPA and cardiovascular outcomes.

Author, Year	Study Design	Sample Size (N)	Age (years)	F (%)	Geographic Location	Baseline SBP/DBP (mmHg)	BMI (kg/m²)	Exposure Definition	Primary Outcome Type	Key Results	Key Covariates Adjusted	Study Quality (NOS)	Follow-up (years)
Vasan (36)	Prospective cohort	1,688 non-hypertensive	M: 55 ± 9	58%	Framingham Heart Study, USA (community)	M: 121/75 ± 10/7	M: 28 ± 4	Serum aldosterone divided into quartiles (Q1 < 9.23, Q2 9.23–12.73, Q3 12.74–17.32, Q4 ≥ 17.32 ng/dL); renin not measured; ARR: not calculated	Incident hypertension	14.8% developed new-onset HTN; 33.6% had increase in BP category.	Age, sex, baseline BP category, systolic BP, diastolic BP, heart rate, BMI, weight gain, diabetes, smoking	9/9 (High)	4
			F: 56 ± 9			F: 117/71 ± 12/8	F: 26 ± 5			17% higher risk of HTN per aldosterone quartile increment.			
										Highest vs. lowest quartile: OR 1.61 (95% CI 1.05–2.46) for incident HTN.			
Markou (17)	Case-control	Cases: 100 normotensives (87 without PA + 13 with PA) Controls: 69 normotensives with normal adrenal CT	53 ± 8	Cases: 80% Controls: NR	Tertiary endocrinology clinic, Greece	118/76 ± 11/8	Without PA: 26 ± 5 With PA: 27 ± 4	ARR ≥0.93 ng/dL·*μ*U/mL AND aldosterone ≥2.96 ng/dL (82 pmol/L); confirmed by fludrocortisone-dexamethasone suppression testing	Incident hypertension	85% of those with normotensive PA (*n* = 13; i.e., 11/13) developed HTN vs. 23% without PA. OR 18.4 (95% CI 3.76–90.10). Note: PA group *n* = 13; estimate based on small denominator.	Age, sex, BMI (limited adjustment given case-control design)	7/9 (High)^a^	5
Brown (13)	Prospective cohort	850 non-HTN: G1: 392 suppressed PRA ≤0.50 G2: 271 intermediate PRA 0.51–0.99 G3: 187 unsuppressed PRA ≥1.0 μg/L/h	G1: 63.3 ± 9.3 G2: 60.4 ± 8.4 G3: 59.7 ± 9.3	G1: 52.8% G2: 44.6% G3: 36.9%	MESA (6 US sites, community-based)	G1: 117.5/73.1 G2: 115.3/71.9 G3: 113.2/71.1	G1: 27.0 ± 4.6 G2: 26.5 ± 4.4 G3: 25.9 ± 4.2	Three PRA phenotypes: suppressed (≤0.50 μg/L/h), intermediate (0.51–0.99 μg/L/h), unsuppressed (≥1.0 μg/L/h); aldosterone modeled as continuous within each phenotype; no fixed binary sPA threshold applied	Incident hypertension	Suppressed renin phenotype with higher aldosterone independently associated with incident HTN. Graded risk relationship across renin phenotypes. All associations independent of baseline BP.	Age, sex, race/ethnicity, BMI, baseline BP, antihypertensive use, smoking, alcohol, diabetes, eGFR	8/9 (High)	10
Hundemer (7)	Prospective cohort	1,284 total: 736 normal BP 548 HTN	54 ± 8	51%	CARTaGENE, Quebec, Canada (community-based)	Normal BP: 113 ± 13/67 ± 9 HTN: 134 ± 14/79 ± 9	∼27	Aldosterone, renin, and ARR modeled as continuous exposures; no fixed binary sPA threshold applied; higher ARR described as indicative of renin-independent aldosteronism	Structural cardiovascular markers (arterial stiffness, cardiac MRI remodeling) and incident hypertension	Higher ARR independently associated with: increased arterial stiffness (central BP, PWV); adverse cardiac MRI remodeling (LVMI, LA volume).	Brachial systolic BP, age, sex, smoking, height, weight, eGFR, serum Na/K, total/HDL/LDL cholesterol, statin use, diabetes, history of CVD, antihypertensive use	8/9 (High)	6.2
										LVH: OR 1.32 (95% CI 1.00–1.73). Incident HTN: OR 1.29 (95% CI 1.03–1.62).			
										All associations independent of brachial BP.			
Goupil (9)	Prospective cohort	2,017 total: 1,472 non-HTN 545 HTN	56 ± 8	45%	CARTaGENE, Quebec, Canada (community-based)	129 ± 15/76 ± 10^c^	NR	Primary: continuous modeling of renin, aldosterone, ARR; *post-hoc* outcome-derived thresholds (not prespecified): renin ≤4.0 ng/L AND ARR ≥70 pmol/L per ng/L	MACE (composite of MI, stroke, HF hospitalization, cardiovascular death)	Lower renin: aHR 2.22 (95% CI 1.02–4.76).	Age, sex, baseline BP, smoking, eGFR, serum Na/K, lipids, statin use, diabetes, history of CVD, antihypertensive use	8/9 (High)^b^	10.8
										Higher ARR: aHR 2.43 (95% CI 1.15–5.12).			
										Post-hoc thresholds: renin ≤4.0 ng/L: aHR 2.12 (95% CI 1.21–3.72); ARR ≥70: aHR 2.03 (95% CI 1.09–3.80).			
										All associations independent of BP.			

sPA, subclinical primary aldosteronism; PA, primary aldosteronism; HTN, hypertension; non-HTN, non-hypertensive; PRA, plasma renin activity (μg/L/h); ARR, aldosterone-to-renin ratio; SBP, systolic blood pressure; DBP, diastolic blood pressure; BMI, body mass index; LVH, left ventricular hypertrophy; LVMI, left ventricular mass index; LA, left atrial; PWV, pulse wave velocity; MACE, major adverse cardiovascular events; MI, myocardial infarction; HF, heart failure; CVD, cardiovascular disease; eGFR, estimated glomerular filtration rate; BP, blood pressure; aHR, adjusted hazard ratio; OR, odds ratio; CI, confidence interval; NR, not reported; M, male; F, female; G, group; MESA, Multi-Ethnic Study of Atherosclerosis. Results are reported as mean ± SD unless otherwise specified (*median and interquartile range).

a Markou et al. scored 7/9 due to clinic-based referral sampling and limited covariate adjustment; the small PA group (*n* = 13) introduces additional statistical instability not captured by the NOS.

b Goupil et al. scored 8/9; the *post-hoc* derivation of biochemical thresholds introduces optimism bias beyond what the NOS captures and requires independent prospective validation.

c Baseline SBP/DBP reflects the mean for the total analytic cohort (*n* = 2,017), which includes both the non-hypertensive (*n* = 1,472) and hypertensive (*n* = 545) subgroups. Mean BP for the non-hypertensive subgroup alone was not reported in the article.

**Table 2:** Study-specific biochemical definitions of sPA with study-specific research criteria.

**Article**	**Exposure Approach**	**Key Methodological Limitation**
Vasan (36)	*Aldosterone quartiles; renin not measured; no ARR.*	Renin-independence of aldosterone secretion cannot be confirmed. Quartile boundaries are not clinical screening cutoffs and are not comparable to ARR- or renin-based definitions in later studies.
Markou (17)	*Binary ARR + aldosterone cutoffs confirmed by formal suppression testing.*	PA group *n* = 13; the 85% conversion rate (11/13) carries wide uncertainty (OR 18.4; 95% CI 3.76–90.10) and cannot be treated as a stable population estimate. Predominantly female clinic-referred cohort limits generalizability.
Baudrand (12)	*PRA <1.0 ng/mL/h entry criterion; urinary aldosterone continuous; 14% met confirmatory PA criteria.*	Prevalence is threshold-dependent: adjusting the suppression test cutoff changes the 14% figure substantially. Confirmatory testing is rigorous but not feasible in routine practice; not comparable to studies using ARR alone.
Brown (13)	*Three PRA phenotype strata; aldosterone modeled continuously within each stratum; no binary sPA threshold.*	Produces no prevalence estimate or clinical decision rule. Risk estimates reflect graded aldosterone differences within renin phenotypes, not a defined sPA category.
Hundemer (7)	*Aldosterone, renin, and ARR all continuous; no binary sPA threshold; structural outcomes by cardiac MRI.*	Per-unit ARR associations cannot identify which individuals to screen or treat. Structural MRI outcomes were cross-sectional at a single time point; prospective threshold validation is lacking.
Goupil (9)	*Primary continuous analysis; post-hoc outcome-derived thresholds: renin ≤4.0 ng/L and ARR ≥70 pmol/L per ng/L.*	Thresholds were derived *post-hoc* from the same dataset used to assess MACE outcomes, introducing optimism bias. They are hypothesis-generating only and require independent prospective validation before clinical application.

Definitions reflect study-specific operational criteria, not consensus diagnostic thresholds for sPA. No standardized diagnostic definition for sPA currently exists.

sPA, subclinical primary aldosteronism; PA, primary aldosteronism; ARR, aldosterone-to-renin ratio; PRA, plasma renin activity; MACE, major adverse cardiovascular events; OR, odds.

The search yielded 1,727 records in total (PubMed/MEDLINE: 902, Web of Science: 318, Cochrane Library: 507). After removal of 307 duplicate records, 1,515 records were screened by title and abstract. Of these, 149 reports were assessed for full-text eligibility, and 144 were excluded due to exclusive focus on overt PA without subclinical characterization, absence of a quantitative aldosterone or renin measure, or reporting of biochemical endpoints without cardiovascular outcomes. Five studies met final inclusion criteria and are summarized in Table 1. The PRISMA flow diagram is presented in Supplementary Figure S1, and the methodological quality of all included studies was assessed using the Newcastle-Ottawa Scale (NOS), with scores reported in Table 1 and detailed results provided in Supplementary Table S1.

## 3 Physiological and pathophysiological role of aldosterone

**Figure 1:**
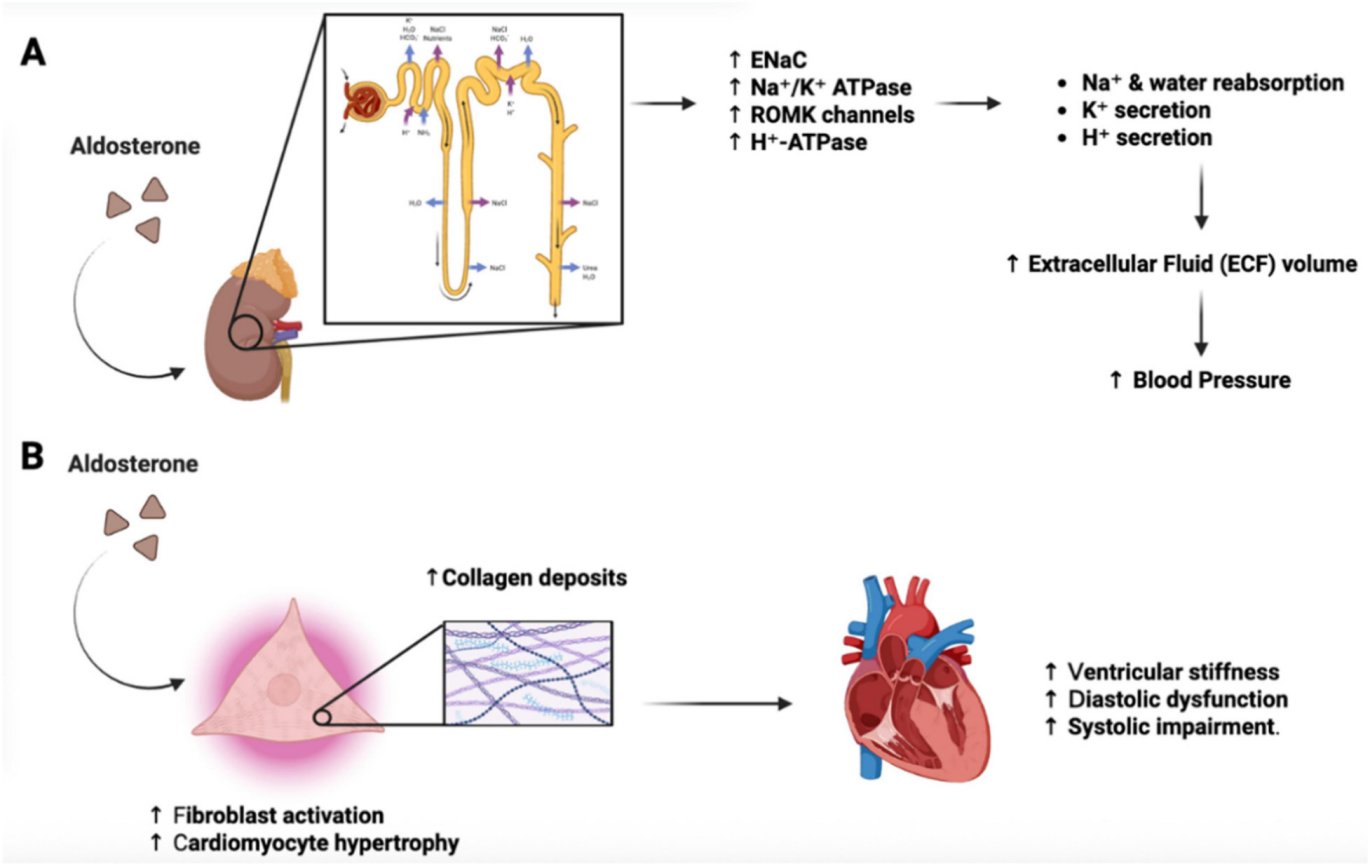
Classical and Non-classical actions of aldosterone in A. Renal and B. Extra-Renal Tissues. **(A) Renal (epithelial) actions.** In the distal nephron and collecting duct, aldosterone binds the mineralocorticoid receptor (MR) and upregulates epithelial sodium channels (ENaC) and Na⁺/K⁺-ATPase, promoting sodium reabsorption and potassium excretion. These actions maintain extracellular volume, electrolyte balance, and acid–base homeostasis. Under normal physiology, this pathway is suppressed by high dietary sodium intake via downregulation of the renin–angiotensin–aldosterone system (23, 24). **(B) ExtrA–Renal (noN,Epithelial) actions.** MRs expressed in cardiomyocytes, vascular smooth muscle cells, endothelial cells, macrophages, and neurons mediate aldosterone's cardiovascular effects independently of its hemodynamic actions. Chronic or inappropriate MR activation in these tissues promotes oxidative stress, vascular inflammation, myocardial and vascular fibrosis through collagen deposition, and structural remodeling eventually leading to increased left ventricular mass, arterial stiffness, and electrophysiologic changes associated with atrial fibrillation. Aldosterone additionally enhances central sympathetic tone and impairs baroreflex sensitivity through central MR activation. (1, 6, 27–30). These pathways are based on mechanistic and observational data. Causal relationships between subclinical aldosteronism and the mentioned outcomes have not been established yet in trials. MR, mineralocorticoid receptor; ENaC, epithelial sodium channel; Na⁺/K⁺-ATPase, sodium-potassium adenosine triphosphatase; LV, left ventricle; AF, atrial fibrillation; RAAS, renin–angiotensin–aldosterone system.

Aldosterone, synthesized in the zona glomerulosa of the adrenal cortex, exerts systemic mineralocorticoid effects through activation of the MR in renal and extra-renal tissues, as seen in Figure 1. Physiologically, its principal action occurs in the distal nephron and collecting duct, where MR activation upregulates epithelial sodium channels (ENaC), promoting sodium reabsorption and potassium excretion and thereby maintaining extracellular volume, electrolyte balance, and acid–base homeostasis (Figure 1A). Although these renal effects are well characterized, aldosterone also acts on cardiovascular, neural, and respiratory tissues through mechanisms that remain incompletely understood (24).

Under conditions of high dietary salt intake, aldosterone secretion is normally suppressed via downregulation of the RAAS; however, in salt-sensitive individuals this feedback inhibition may be blunted, resulting in inappropriately elevated aldosterone levels despite sodium excess (25). This maladaptive response may contribute to impaired natriuresis, volume expansion, and sustained cardiovascular stress (26). Notably, overt aldosterone excess has also been associated with altered sodium chloride taste perception in affected patients, a finding that may have implications for dietary behavior and salt intake (26).

Chronic inappropriate MR activation has been associated with inflammation, oxidative stress, and fibrosis within the myocardium and vasculature, leading to collagen deposition, structural remodeling, and increased left ventricular mass, as shown in Figure 1B (23, 27). Experimental evidence also suggests that aldosterone-induced cardiac fibrosis occurs in both ventricles, extending beyond the left ventricle and thus appearing to be at least partly independent of hemodynamic load and elevated blood pressure (28). Furthermore, mineralocorticoid excess has also been associated with increased left ventricular mass index and left ventricular hypertrophy, atrial enlargement, and arterial stiffness as reflected by elevated central blood pressure and pulse wave velocity (7). Aldosterone additionally influences autonomic regulation through central MR activation, and has been shown to impair baroreflex sensitivity and enhance sympathetic tone in both experimental and clinical studies (24, 29, 30).

Clinically, these changes may increase susceptibility to hypertension, cardiomyopathy, and atrial fibrillation through profibrotic and electrophysiologic remodeling mechanisms (1, 6). Moreover, excess aldosterone has been implicated in obstructive sleep apnea via fluid redistribution in the supine position and through upper airway muscle dysfunction and collapse (31).

Collectively, these physiological and pathophysiological effects position aldosterone as a critical regulator of cardiovascular and systemic homeostasis whose dysregulation have been associated with cardiometabolic disease across multiple organ systems. Importantly, associations between aldosterone excess and structural cardiovascular abnormalities have been observed in normotensive individuals in population-based cohort data, suggesting tissue-level MR effects may extend beyond blood pressure-mediated injury (7, 32).

## 4 The Spectrum of renin-independent aldosteronism

PA is a well-established syndrome characterized by autonomous and inappropriately elevated aldosterone secretion from one or both adrenal glands, leading to chronically unregulated MR activation. Increasing evidence builds on foundational conceptual work set over the past two decades, supporting the view that PA exists along a biological continuum rather than as a binary disorder defined solely by conventional diagnostic thresholds (9, 12, 16).

The literature in this field uses several related but non-identical terms that are worth clarifying before proceeding. The broadest construct, the renin-independent aldosterone excess, refers to a research phenotype in which plasma renin activity is suppressed and aldosterone secretion is relatively non-suppressible, even in the absence of a confirmed PA diagnosis and even among normotensive individuals (12, 13). Narrower than this are the study-specific definitions of sPA, which differ substantially across cohorts in their choice of ARR cutoffs, renin thresholds, and whether confirmatory suppression testing was performed; these are summarized in Table 2. At the far end of the spectrum sits overt PA, defined by the Endocrine Society as requiring biochemical confirmation through recognized suppression tests (11, 23). Throughout this review, the term sPA is used to denote biochemical phenotypes that fall below these confirmatory thresholds, acting as a working definition that reflects the current absence of consensus criteria rather than an established diagnostic category.

Suppression of renin concentration or activity may indicate renin-independent aldosterone secretion even when circulating aldosterone levels are only modestly elevated (12). This paradigm has led to the recognition of sPA, also referred to as nonclassical renin-independent aldosterone excess (7, 16). Biochemically, sPA is characterized by dysregulated aldosterone production in the context of suppressed renin that does not fulfill traditional criteria for overt PA (7, 16). Emerging data suggest that biochemical phenotypes consistent with sPA are both prevalent and substantially underdiagnosed (7). Several factors contribute to this underdiagnosis. Many patients with this biological phenotype lack the classical features that trigger clinical suspicion, such as hypokalemia that is absent in the majority of patients (33). Even when screening is pursued, traditional ARR thresholds carry substantial intraindividual variability and may fail to capture mild or intermittent aldosterone excess (34). On top of all this, is the absence of consensual diagnostic criteria for sPA itself, as unlike overt PA there is no agreed biochemical threshold that defines the condition or identifies which populations should be screened (11, 22, 23, 33). Hence, quantifying the prevalence of a condition that exists along a physiological spectrum presents inherent methodological challenges (13, 16, 35).

In current clinical practice, screening for PA is frequently initiated only after the development of associated comorbidities, such as resistant hypertension, hypokalemia, atrial fibrillation, or adrenal incidentaloma, rather than at a preclinical stage (21). This reactive strategy contrasts with the foundational epidemiologic principle of screening, which aims to identify disease in its preclinical stage. Because hypertension may represent a downstream manifestation of aldosterone excess rather than its earliest expression, this raises the hypothesis that earlier biochemical evaluation of renin and aldosterone profiles in normotensive or mildly hypertensive individuals might improve cardiovascular risk stratification (9, 12, 13). This hypothesis, while biologically plausible, still requires prospective validation before it can inform clinical practices.

## 5 Role of subclinical primary aldosteronism in cardiovascular disease

The accumulating observational evidence suggests that biochemical phenotypes consistent with sPA may represent independently associated cardiovascular risk factors, fundamentally challenging the traditional view of aldosterone excess as solely a cause of overt hypertension. A compelling narrative, through prospective studies in normotensive and early stage hypertensive patients (Table 1), suggested a continuum of non-suppressible renin-independent aldosterone secretion that may precede clinical PA, progression to hypertension, or the onset of major cardiovascular events (2, 7, 9, 12, 13, 36).

The earliest evidence of aldosterone's insidious role came from prospective studies linking seemingly “normal” aldosterone levels to future hypertension. As early as 2004, Vasan et al. showed that higher baseline aldosterone, even within the normal range, was associated with higher incident hypertension in normotensive and early stages hypertensive individuals (36). Specifically, in this longitudinal study over four years, 14.8% of participants developed incident hypertension and 33.6% experienced an increase in blood pressure category (36). Each quartile increment in serum aldosterone within the physiologic range was independently associated with a 17% higher risk of incident hypertension, with those in the highest aldosterone quartile carrying a 1.61-fold higher risk compared with those in the lowest quartile (95% CI 1.05–2.46), suggesting a graded dose-response relationship (36). However, a key limitation of this study is the absence of plasma renin measurement, which prevents confirmation of the renin-independent nature of the observed aldosterone associations, hence limiting the ability to distinguish autonomous from physiologic aldosterone secretion. Ten years later, Markou et al. reinforced this in a case-control study with a study-specific biochemical definition of sPA (Table 2), demonstrating that 85% of patients with normotensive PA develop hypertension over 5 years, compared to 23% of those without normotensive PA (OR 18.4; 95% CI 3.8–90.1) (17). However, this study included a small number of PA cases and hence requires caution when interpreting those results (17). However, the small PA group size (*n* = 13) in this study produces wide confidence intervals and limits the precision and generalizability of this OR. Subsequently, in a larger MESA cohort study of 850 untreated normotensive participants, Brown et al. found that a suppressed renin phenotype combined with higher aldosterone levels was independently associated with increased risk of incident hypertension over ten years, hence suggesting a graded risk relationship across renin phenotypes (13). A main limitation of this study is that hormonal phenotyping relied on a single measurement under *ad libitum* sodium conditions, which may not reflect sustained renin suppression. On the other hand, Sartori et al. observed that among hypertensive individuals without a confirmed PA diagnosis, higher aldosterone levels were associated with greater likelihood of developing resistant hypertension (37). Taken together, these findings suggest that individuals with biochemical sPA phenotypes may represent not merely a cardiovascular risk factor but an early point on a trajectory toward resistant hypertension, though whether intervention at this stage would alter the trajectory remains untested.

**Figure 2:**
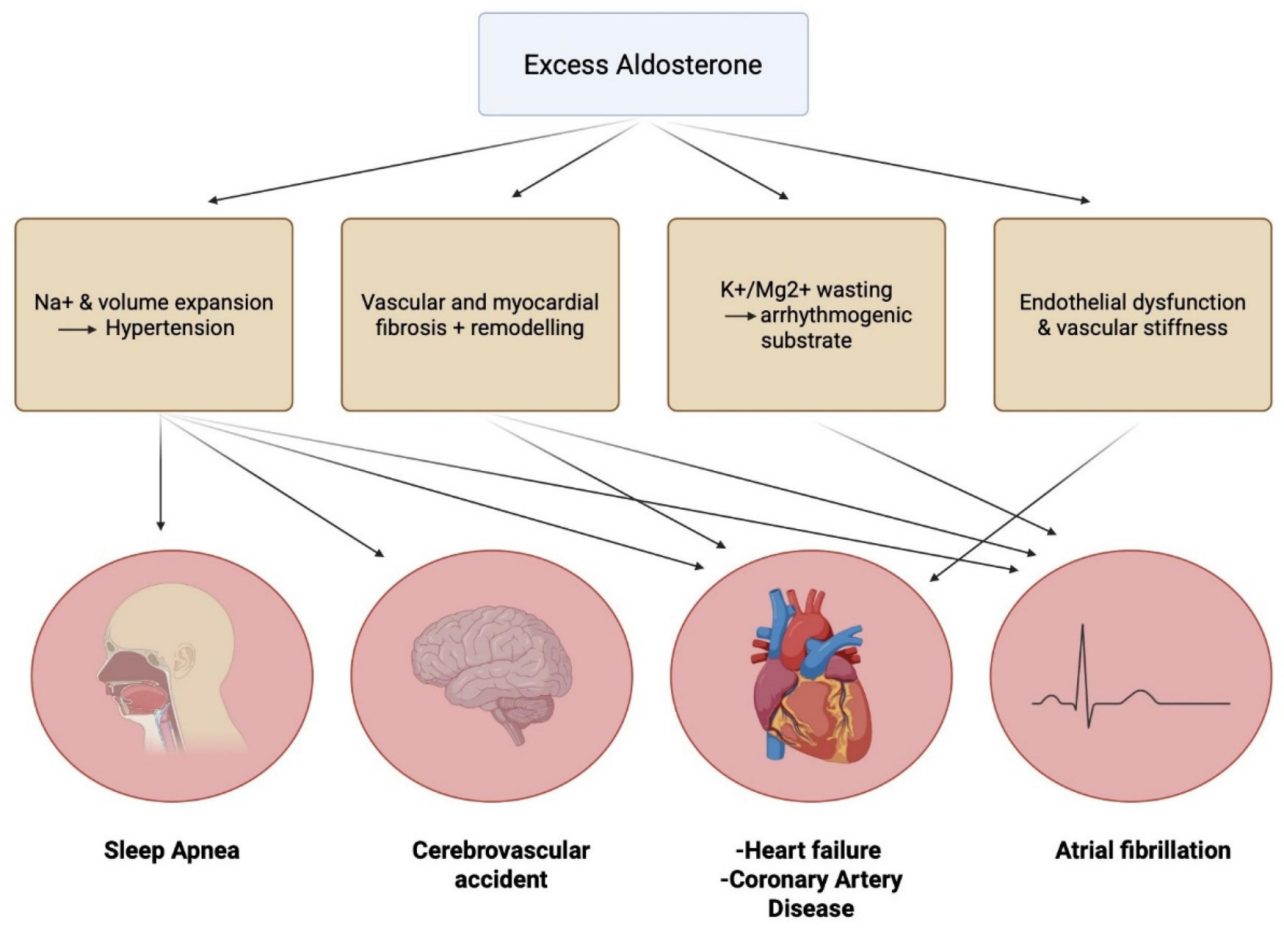
Proposed pathophysiological pathways linking subclinical primary aldosteronism to cardiovascular disease.

Beyond blood pressure, biochemical sPA phenotypes have been associated with direct cardiovascular damage, independent of hypertension, highlighting its possible systemic implications. Two prospective analyses from the population-based CARTaGENE cohort in Quebec provide the most detailed evidence to date, one examining subclinical structural endpoints, the other hard clinical outcomes (7, 9) The proposed pathways linking renin-independent aldosterone excess to downstream cardiovascular outcomes are summarized in Figure 2.

Hundemer et al. enrolled 1,284 adults aged 40–69 and utilized aldosterone, renin, and ARR as continuous exposures without applying a binary sPA threshold (7). In the 736 participants with normal blood pressure at baseline, higher ARR was independently associated with increased arterial stiffness by pulse wave velocity, adverse cardiac remodeling on MRI including elevated left ventricular mass index and left atrial volume (7). Moreover, higher ARR was also independently associated with greater odds of left ventricular hypertrophy (OR 1.32; 95% CI 1.00–1.73) and incident hypertension (OR 1.29; 95% CI 1.03–1.62) (7). All those associations were independent of brachial blood pressure (7). A significant caveat to this is that arterial stiffness outcomes were merely cross-sectional hence decreasing their strength of association.

Goupil et al. used the same cohort with a larger sample (*n* = 2,017) and longer follow-up of approximately 10.8 years, linking baseline biochemistry to hard MACE outcomes (9). Again using continuous modeling as the primary approach, lower renin and higher ARR were independently associated with greater MACE risk. A *post-hoc* step then derived outcome-optimized thresholds: renin ≤4.0 ng/L and ARR ≥ 70 pmol/L per ng/L were each associated with approximately twofold higher risk of MACE, independent of blood pressure, and yielding an adjusted hazard ratio of approximately 2.4 (9). These thresholds were not prespecified but identified retrospectively from the outcome data, which limits their applicability as prospective screening cutoffs without independent validation.

Longitudinal data further suggest that individuals with biochemical sPA phenotypes may accumulate cardiovascular injury over time even before a formal PA diagnosis is established, and that this injury is detectable at subclinical stages in population-based cohorts (3, 7, 9, 23). Such progression results in a prolonged exposure of the body to inappropriately high aldosterone levels, which may underlie the development of incident hypertension, atrial fibrillation, and coronary disease in PA which further supports the concept that even mild elevations in aldosterone are not benign (9).

The accumulating observational evidence suggests a dose-dependent relationship between the degree of renin-independent aldosterone excess and cardiovascular risk, spanning from subclinical structural remodeling to incident MACE (7, 9, 13, 36). These observations raise the hypothesis that screening strategies may need to move beyond identifying overt PA toward detecting earlier renin-suppressed phenotypes, with the aim of informing prospective studies examining whether earlier identification reduces long-term cardiovascular burden (12, 13). The biochemical thresholds associated with adverse outcomes in current observational data are summarized in Tables 1, Table 2. However, whether these thresholds should guide clinical action requires prospective interventional validation that is currently lacking.

## 6 Biochemical diagnosis of sPA and threshold for cardiovascular disease

No consensus biochemical threshold for sPA has been established, as summarized in Table 2. The diagnostic landscape for sPA is complicated by three overlapping challenges: the absence of a standardized sPA definition, the known limitations of available screening tests when applied outside the classical PA phenotype, and the lack of prospective validation linking any specific biochemical threshold to cardiovascular benefit from intervention. The following subsections address each marker in turn.

### 6.1 ARR and/or other parameters

Several studies have explored ARR and related biochemical markers in normotensive populations. Markou et al. identified a biochemical profile consistent with PA in normotensive individuals using ARR and aldosterone cutoffs substantially below conventional PA screening thresholds, with those meeting criteria carrying a markedly elevated risk of incident hypertension (17). Similarly, in a cohort of 736 normotensive individuals, Hundemer et al. found that higher ARR values, treated as a continuous exposure, were associated with markers of cardiovascular injury (7). Notably, this analysis evaluated aldosterone and renin as continuous variables and do not propose specific threshold above which cardiovascular risk begins to rise (7). In contrast, Goupil et al. went further, deriving *post-hoc* thresholds for specific outcomes (9). The proposed biochemical thresholds included renin concentration ≤4.0 ng/L and ARR ≥70 pmol/L per ng/L, hence identifying a subgroup of normotensive individuals with disproportionately elevated cardiovascular risk (9). Individuals meeting both criteria had a 2.4-fold increased risk of MACE (aHR 2.42; 95% CI, 1.25–6.48) compared with those with higher renin levels and lower ARR values (9). Notably, the Goupil thresholds are considerably less stringent than the standard ES guideline renin threshold of ≤1.0 ng/L used for overt PA screening, suggesting that meaningful aldosterone-mediated cardiovascular risk may occur at renin levels well above current screening triggers (9, 11). However, these thresholds were derived retrospectively from the same dataset in which outcomes were assessed, hence increasing the risk of overfitting to cohort-specific characteristics. These thresholds should be considered strictly as hypothesis-generating and require rigorous external validation in independent prospective cohorts before any consideration for clinical use.

### 6.2 Aldosterone

Rare studies used aldosterone alone in the classification of PA and sPA (36). Studies using urine aldosterone cutoff of ≥10 mcg/24 h suggest that PA is not solely related to resistant hypertension (20% in normotensives, 27% in stage 1 hypertensives, 40% in stage 2 hypertensives, and 60% among resistant hypertensives) (13, 38). The ascending percentages might suggest that aldosterone excess in patients with normotension may progress into hypertension. Importantly, serum aldosterone is prone to be influenced by many factors, such as plasma potassium levels, with even small potassium shifts affecting test accuracy, hence limiting its reliability as a standalone screening marker (23).

### 6.3 Renin

Plasma renin activity was used alongside other biomarkers to identify sPA, though some studies relied on it alone as a standalone marker. In a cohort study using plasma renin activity cutoff of ≤1 ng/mL/h to define renin-independent aldosterone secretion, 14% of normotensive individuals with suppressed renin phenotype were found to have biochemically confirmed PA with only 21% of this population have ARR ≥ 20 (12). This illustrates that suppressed renin alone potentially detects a meaningfully distinct population than ARR-based screening.

ARR has long been utilized as the initial screening test for PA. While Hiramastu et al. first introduced ARR as a diagnostic tool in 1981, its clinical utility remains controversial due to variable performance across studies (15). In a meta-analysis of 4,110 patients, Hung et al. found that ARR sensitivity ranged from 10% to 100% across studies, with three of ten studies reporting sensitivity below 50%, rendering the determination of a definitive optimal ARR cutoff unfeasible (39). A retrospective study further demonstrated substantial variability in aldosterone, renin, and ARR values on repeat testing, even after correcting for potassium levels and withdrawal of antihypertensive medications (34). Furthermore, significant intraindividual variability in ARR with a coefficient of variation of up to 45% has been reported (34). Several physiological factors can also influence ARR measurements, including salt intake, posture, time of day, menstrual cycle phase, and serum potassium levels (33). Despite these limitations, ARR remains widely used due to its convenience and cost-effectiveness. However, guidelines recommend repeating the test when results do not align with the clinical presentation. ARR use in sPA, especially in case of normotension, would be challenging as ARR sensitivity is significantly low, ranging from 21% to 27% using cutoffs of >20 and >30 ng/dL per ng/mL/h, respectively (34, 40).

Aldosterone secretion itself shows diurnal variability, which can further complicate interpretation. A single (spot) serum aldosterone measurement may not reliably rule out primary aldosteronism because aldosterone levels fluctuate throughout the day, making 24-hour urine aldosterone more reliable (41). In a large retrospective cohort of 94,829 patients, Marcelli et al. showed that screening based on PRA < 1 ng/mL/h alone identified 25.6% more patients compared to using ARR ≥ 20. In granular detail, 45.9% of patients with PA tested positive when assessed through suppressed renin (<1 ng/mL/h) vs. 20.3% and 13.9% of patients having PA tested positive based on ARR ≥ 20 and ARR ≥ 30, respectively. When using ARR ≥ 20 or ≥30 as screening test, almost all (96.7% and 98.7%) had a suppressed PRA, however, only 42.8% and 29.8% in the suppressed renin group had an ARR using ≥20 or ≥30 (40). This asymmetry suggests that PRA suppression captures a substantially broader at-risk population than ARR alone.

**Figure 3:**
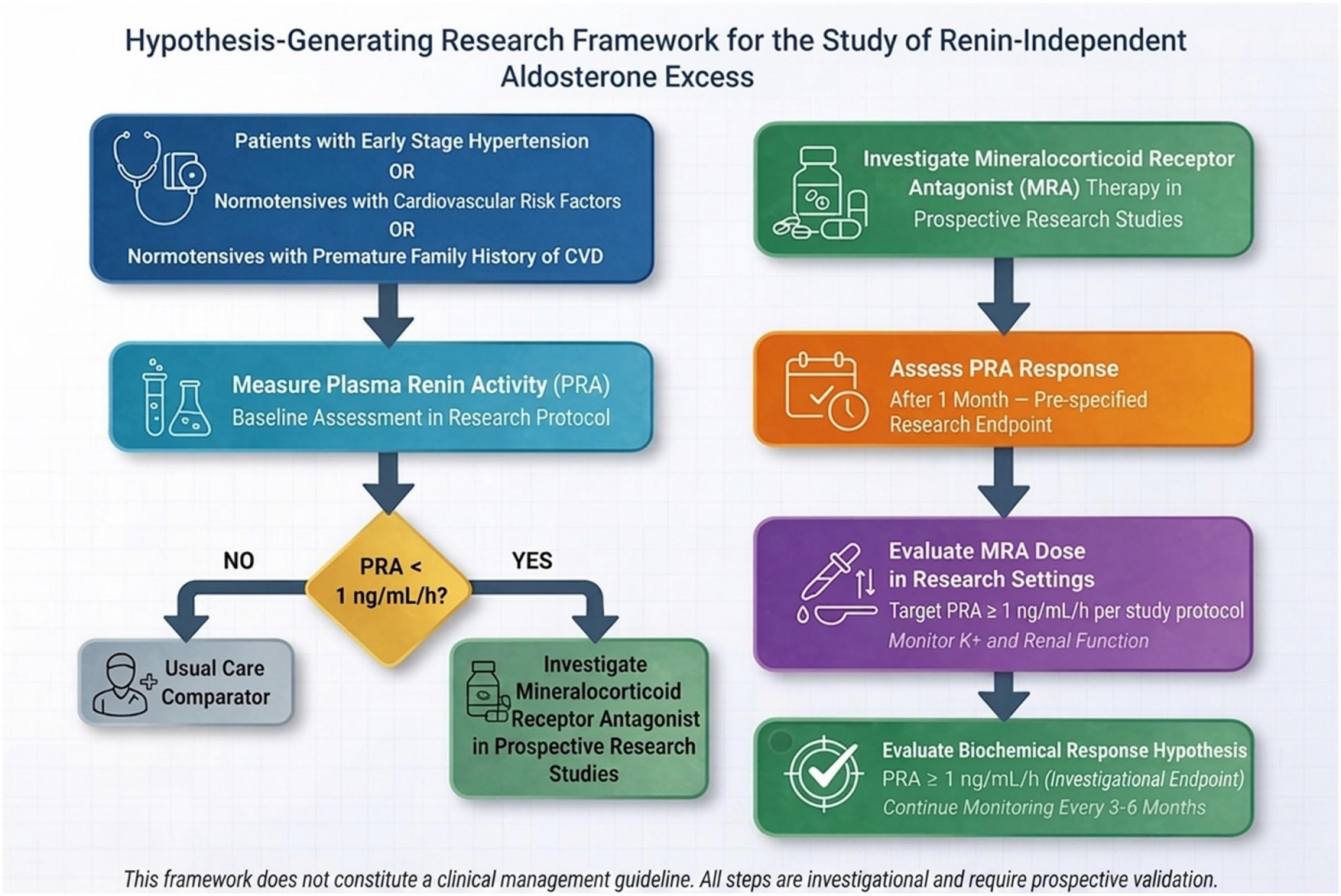
Hypothesis-Generating research framework for the study of renin-independent aldosterone excess.

Given this evidence, PRA suppression (<1 ng/mL/h) may represent a more sensitive, though less specific, indicator of renin-independent aldosteronism than ARR when screening for sPA, particularly in normotensive and mildly hypertensive populations where ARR sensitivity is lowest (40). Whether this observation should translate to a revised screening strategy requires prospective evaluation, given that current data are insufficient to support a specific clinical recommendation. A proposed hypothesis-generating research framework for future investigational studies is presented in Figure 3.

## 7 Physiologic effect of anti- renin-angiotensin-aldosterone system (RAAS) medication on heart function

Before reviewing the evidence, it is important to acknowledge the central limitation of this section, as the overwhelming majority of existing MRA data derives from populations with overt PA, heart failure with reduced ejection fraction (HFrEF), or heart failure with preserved ejection fraction (HFpEF), not from individuals with sPA. Hence, translating therapeutic findings from these populations to sPA requires caution.

### 7.1 Tier 1: mechanistic rationale for MRA therapy

While the cardiovascular damage associated with aldosteronism is mainly mediated by excessive and chronic activation of the MR in cardiac and vascular tissues (13, 23), the beneficial effects of MRAs in cardiovascular diseases arise from a range of complex and multifaceted mechanisms (27). For example, trials have demonstrated the efficacy of MRAs in reducing biomarker of fibrosis when given after myocardial infarction and stroke (42, 43). Moreover, clinical studies showed that aldosterone blockade improve neck girth significantly in patients with obstructive sleep apnea (44). These observations establish a plausible rationale for MRA therapy wherever excess aldosterone-driven MR activation contributes to tissue injury.

### 7.2 Tier 2: indirect supportive evidence from adjacent populations

Pharmacological treatment with MRAs such as spironolactone and eplerenone is currently the most effective pharmacological treatment for bilateral PA, as studies have demonstrated that MRAs lower blood pressure, reverse left ventricular hypertrophy and intracardiac volume, improve diastolic function, reduce arterial stiffness, and lower albuminuria (23, 45, 46). It is crucial to note that their benefit extends even beyond blood pressure regulation (27), as they were also found to affect atrial fibrillation (AF) (47). Regarding atrial fibrillation, adrenalectomy in lateralized PA has been associated with reduced new-onset AF incidence, however, the evidence for MRAs in this context is less consistent, with some studies showing benefit and others showing neutral results in the PA population specifically (47). In broader structural heart disease populations, MRAs have been associated with reduced AF-related hospitalization and maintenance of sinus rhythm, though these benefits are not established specifically in PA or sPA patients (48, 49). Nevertheless, this evidence is derived from overt bilateral PA populations and cannot be generalized to the sPA biochemical phenotype without prospective validation.

### 7.3 Extrapolation: not evidence in sPA

Beyond PA populations, MRAs have demonstrated cardiovascular benefit in heart failure populations. In HFpEF, the TOPCAT trial showed reduced heart failure hospitalizations with spironolactone, though the primary composite endpoint was not met (50). In HFrEF patients, the RALES and EMPHASIS-HF trials established MRAs as life-prolonging therapies, significantly reducing mortality and rehospitalization (50–52). These findings establish a mechanistic basis for MR blockade in aldosterone-driven cardiovascular injury but all of which were derived from populations with established structural heart disease, not from individuals with subclinical biochemical aldosterone excess. Extrapolation to sPA biochemical phenotypes should therefore be made with caution.

### 7.4 Tier 3: direct evidence in sPA

While sPA is considered a milder biochemical phenotype along the PA spectrum, it still exposes the body to inappropriately elevated aldosterone levels, and has been associated with a spectrum of silent but potentially harmful effects. As a largely asymptomatic hormonal dysregulation, and in the absence of definitive randomized controlled trials demonstrating that early intervention prevents long-term MACE, sPA phenotype construct remains an evolving area of investigation. To date, most of the existing evidence has focused on overt primary aldosteronism. Emerging studies suggest that targeted medical interventions may help reduce some cardiovascular risks, such as arterial stiffness and left ventricular hypertrophy, associated with aldosterone excess in primary aldosteronism (45, 53). Although most studies do not classify patient with PA into mild or severe form, treatment strategies for normotensive or asymptomatic patients with the sPA phenotype are still under debate and not yet well defined (2). One small study in which mild PA was diagnosed based on low PRA, 25% of this population exhibiting an ARR < 20 ng/dL per ng/mL/h, treatment with a MRA reduced blood pressure by 12 mmHg (from a baseline of 137 mmHg to 125 mmHg) (54). No randomized trial has prospectively evaluated whether MRA therapy in sPA prevents incident hypertension, structural remodeling, or MACE.

Taken together, the rationale and indirect evidence from adjacent populations support the biological plausibility of MRA benefit in sPA. However, the absence of sPA-specific randomized trial data means that therapeutic recommendations cannot be made at this time. This represents an important gap that prospective trials are needed to address.

## 8 Conclusion

Primary aldosteronism likely represents a clinical continuum in which renin-independent aldosterone excess, even below conventional diagnostic thresholds, has been associated with arterial stiffness, adverse cardiac remodeling, incident hypertension, and major adverse cardiovascular events independent of blood pressure, as observed in population-based cohort data. These associations are biologically plausible and consistent across studies but remain observational.

Several limitations reduce the clinical implications of these findings. Currently, no consensual diagnostic threshold for sPA exists, as biochemical definitions vary substantially across cohorts, and aldosterone and renin measurements are very sensitive to pre-analytic conditions that limit reproducibility in routine practice. Available therapeutic evidence derives from overt PA and heart failure populations rather than from sPA directly, and no randomized trial has demonstrated that identifying or treating sPA improves hard cardiovascular outcomes. Specifically, no randomized trial has evaluated MRA therapy in individuals with sPA biochemical phenotype, therefore no treatment recommendation for this population can currently be made. The *post-hoc* derivation of the Goupil thresholds introduces optimism bias and precludes their use as validated clinical screening cutoffs pending independent prospective replication. Furthermore, the two CARTaGENE studies providing the strongest outcome data were drawn from a predominantly White, French-Canadian population (approximately 92%), which may limit generalizability to more ethnically diverse populations in whom the prevalence and phenotype of sPA may differ.

Prospective cohort studies using standardized biochemical definitions and pre-specified endpoints are needed to characterize the natural history of sPA biochemical phenotype. Pragmatic clinical trials testing renin-guided screening and early mineralocorticoid receptor antagonism in normotensive and mildly hypertensive individuals are ultimately required to determine whether intervening at the subclinical stage translates into meaningful reductions in long-term cardiovascular harm.

The biochemical phenotype of subclinical primary aldosteronism (sPA) is characterized by renin-independent aldosterone excess that falls below conventional diagnostic thresholds for overt PA. Through chronic and inappropriate mineralocorticoid receptor (MR) activation in cardiac, vascular, neural, and respiratory tissues, aldosterone excess initiates several converging pathophysiological cascades.

At the myocardial and vascular level, MR activation promotes oxidative stress, vascular inflammation, and collagen deposition, leading to myocardial fibrosis and structural remodeling. These changes manifest as increased left ventricular mass index (LVMI), left ventricular hypertrophy (LVH), left atrial enlargement, and elevated arterial stiffness reflected by increased pulse wave velocity (PWV) and central blood pressure. Profibrotic and pro-arrhythmogenic remodeling of atrial tissue contributes to the development of atrial fibrillation (AF) (21, 27, 28, 42, 43).

At the hemodynamic level, aldosterone-driven sodium retention and volume expansion promote incident hypertension, which in turn accelerates cardiovascular remodeling and increases the risk of coronary artery disease (CAD) and cerebrovascular events (CVA/stroke) (7, 13, 36).

Through central MR activation, aldosterone enhances sympathetic tone and impairs baroreflex sensitivity, further amplifying blood pressure instability and arrhythmia risk. Through fluid redistribution and upper airway muscle dysfunction, aldosterone excess is additionally associated with obstructive sleep apnea (OSA), which independently contributes to hypertension and cardiovascular morbidity (24, 29–31, 44).

The cumulative downstream consequence of these converging pathways is an elevated risk of major adverse cardiovascular events (MACE), including myocardial infarction, stroke, heart failure, and cardiovascular death as observed in population-based longitudinal cohort data even below conventional PA diagnostic thresholds (7, 9).

Pathways depicted are based on mechanistic, observational, and population-based cohort data. Causal directionality has not been established in randomized controlled trials. Abbreviations: sPA, subclinical primary aldosteronism; PA, primary aldosteronism; MR, mineralocorticoid receptor; LVMI, left ventricular mass index; LVH, left ventricular hypertrophy; PWV, pulse wave velocity; AF, atrial fibrillation; CAD, coronary artery disease; CVA, cerebrovascular accident; OSA, obstructive sleep apnea; MACE, major adverse cardiovascular events; BP, blood pressure; HTN, hypertension; HF, heart failure.

This model illustrates a hypothesis-generating framework for future studies evaluating whether identification of subclinical primary aldosteronism, manifesting as suppressed renin phenotypes, and subsequent mineralocorticoid receptor antagonist therapy may modify biochemical or cardiovascular outcomes. The figure is intended for research conceptualization and does not represent a clinical management algorithm. CVD: cardiovascular diseases; MRA: Mineralocorticoid Receptor Antagonist; PRA: Plasma Renin Activity. The PRA threshold of <1 ng/mL/h used in this framework is derived from Baudrand et al. and Marcelli et al. and reflects a study-specific research criterion. Those thresholds are not a validated and should not be used as a clinical screening cutoff and should be interpreted only as hypothesis-generating (7, 9, 12, 39, 40, 54).

## Supplementary material

Supplementary Figure S1 PRISMA flow diagram of literature search and study selection.

Supplementary Table S1 Newcastle-Ottawa Scale quality assessment of included studies.

## References

[1] Ekman N Grossman AB Nieckarz A Jędrzejewski Ł Wolf J Dworakowska D. Non-hypertensive effects of aldosterone. Int J Mol Sci. (2025) 26(2):540. 10.3390/ijms26020540 39859256 PMC11766190

[2] Young MJ Clyne CD. Mineralocorticoid receptor actions in cardiovascular development and disease. Essays Biochem. (2021) 65(6):901–11. 10.1042/EBC20210006 34414409

[3] Rocha R Rudolph AE Frierdich GE Nachowiak DA Kekec BK Blomme EAG. Aldosterone induces a vascular inflammatory phenotype in the rat heart. Am J Physiol Heart Circ Physiol. (2002) 283:H1802–10. 10.1152/ajpheart.01096.2001 12384457

[4] Rickard AJ Young MJ. Corticosteroid receptors, macrophages and cardiovascular disease. J Mol Endocrinol. (2009) 42:449–59. 10.1677/JME-08-0144 19158233

[5] Bienvenu LA Morgan J Rickard AJ Tesch GH Cranston GA Fletcher EK. Macrophage mineralocorticoid receptor signaling plays a key role in aldosterone-independent cardiac fibrosis. Endocrinology. (2012) 153:3416–25. 10.1210/en.2011-2098 22653557

[6] Otsuka H Abe M Kobayashi H. The effect of aldosterone on cardiorenal and metabolic systems. Int J Mol Sci. (2023) 24(6):5370. 10.3390/ijms24065370 36982445 PMC10049192

[7] Hundemer GL Agharazii M Madore F Vaidya A Brown JM Leung AA. Subclinical primary aldosteronism and cardiovascular health: a population-based cohort study. Circulation. (2024) 149(2):124–34. 10.1161/CIRCULATIONAHA.123.066389 38031887 PMC10841691

[8] Huang M Li J Zhao X Fu R Li X Jiang W. Global and regional prevalence and cardiovascular risk of primary aldosteronism: a systematic review and meta-analysis. Curr Probl Cardiol. (2024) 49(10):102791. 10.1016/j.cpcardiol.2024.102791 39127431

[9] Goupil R Desbiens L-C Merabtine A Agharazii M Madore F Vaidya A. Subclinical primary aldosteronism and Major adverse cardiovascular events: a longitudinal population-based cohort study. Circulation. (2025) 152(13):913–23. 10.1161/CIRCULATIONAHA.124.073507 40631720 PMC12243960

[10] Ito Y Takeda R Takeda Y. Subclinical primary aldosteronism. Best Pract Res Clin Endocrinol Metab. (2012) 26(4):485–95. 10.1016/j.beem.2011.11.006 22863390

[11] Adler GK Stowasser M Correa RR Khan N Kline G McGowan MJ. Primary aldosteronism: an endocrine society clinical practice guideline. J Clin Endocrinol Metab. (2025) 110(9):2453–95. 10.1210/clinem/dgaf284 40658480

[12] Baudrand R Guarda FJ Fardella C Hundemer G Brown J Williams G. Continuum of renin-independent aldosteronism in normotension. Hypertension. (2017) 69(5):950–6. 10.1161/HYPERTENSIONAHA.116.08952 28289182 PMC5391287

[13] Brown JM Robinson-Cohen C Luque-Fernandez MA Allison MA Baudrand R Ix JH. The spectrum of subclinical primary aldosteronism and incident hypertension. Ann Intern Med. (2017) 167(9):630–41. 10.7326/M17-0882 29052707 PMC5920695

[14] Adlin EV. Plasma renin and blood pressure. Lancet. (1975) 305:699. 10.1016/S0140-6736(75)91814-0 47134

[15] Hiramatsu K Yamada T Yukimura Y Komiya I Ichikawa K Ishihara M. A screening test to identify aldosterone-producing adenoma by measuring plasma renin activity. Arch Intern Med. (1981) 141:1589–93. 10.1001/archinte.1981.00340130033011 7030245

[16] Turcu AF Yang J Vaidya A. Primary aldosteronism: a multidimensional syndrome. Nat Rev Endocrinol. (2022) 18(11):665–82. 10.1038/s41574-022-00730-2 36045149

[17] Markou A Pappa T Kaltsas G Gouli A Mitsakis K Tsounas P. Evidence of primary aldosteronism in normotensive individuals: a very high odds ratio for progression into arterial hypertension. J Clin Endocrinol Metab. (2013) 98(4):1409–16. 10.1210/jc.2012-3353 23471976

[18] Nishimoto K Tomlins SA Kuick R Cani AK Giordano TJ Hovelson DH. Aldosterone-stimulating somatic gene mutations are common in normal adrenal glands. Proc Natl Acad Sci U S A. (2015) 112(33):E4591–9. 10.1073/pnas.1505529112 26240369 PMC4547250

[19] Omata K Tomlins SA Rainey WE. Aldosterone-producing cell clusters in normal and pathological states. Horm Metab Res. (2017) 49(12):951–6. 10.1055/s-0043-122394 29202494 PMC5770278

[20] Cohen JB Cohen DL Herman DS Leppert JT Byrd JB Bhalla V. Testing for primary aldosteronism and mineralocorticoid receptor antagonist use among US veterans: a retrospective cohort study. Ann Intern Med. (2021) 174:289–97. 10.7326/M20-4873 33370170 PMC7965294

[21] Hundemer GL Imsirovic H Vaidya A Yozamp N Goupil R Madore F. Screening rates for primary aldosteronism among individuals with hypertension plus hypokalemia: a population-based retrospective cohort study. Hypertension. (2022) 79:178–86. 10.1161/HYPERTENSIONAHA.121.18118 34657442 PMC8664996

[22] Liu Y- King J Kline GA Padwal RS Pasieka JL Chen G. Outcomes of a specialized clinic on investigation and treatment of primary aldosteronism. JAMA Surg. (2021) 156:541–9. 10.1001/jamasurg.2021.0254 33787826 PMC8014194

[23] Funder JW Carey RM Mantero F Murad MH Reincke M Shibata H. The management of primary aldosteronism: case detection, diagnosis, and treatment: an endocrine society clinical practice guideline. J Clin Endocrinol Metab. (2016) 101:1889–916. 10.1210/jc.2015-4061 26934393

[24] Johnston JG Welch AK Cain BD Sayeski PP Gumz ML Wingo CS. Aldosterone: renal action and physiological effects. Compr Physiol. (2023) 13(2):4409–91. 10.1002/j.2040-4603.2023.tb00256.x 36994769 PMC11472823

[25] Drenjančević-Perić I Jelaković B Lombard JH Kunert MP Kibel A Gros M. High-salt diet and hypertension: focus on the renin-angiotensin system. Kidney Blood Press Res. (2011) 34(1):1–11. 10.1159/000320387 21071956 PMC3214830

[26] Adolf C Görge V Heinrich DA Hoster E Schneider H Handgriff L. Altered taste perception for sodium chloride in patients with primary aldosteronism. Hypertension. (2021) 77(4):1332–40. 10.1161/HYPERTENSIONAHA.120.16440 33641355

[27] Jaisser F Farman N. Emerging roles of the mineralocorticoid receptor in pathology: toward new paradigms in clinical pharmacology. Pharmacol Rev. (2016) 68:49–75. 10.1124/pr.115.011106 26668301

[28] Robert V Van Thiem N Cheav SL Mouas C Swynghedauw B Delcayre C. Increased cardiac types I and III collagen mRNAs in aldosterone-salt hypertension. Hypertension. (1994) 24:30–6. 10.1161/01.HYP.24.1.30 8021005

[29] Pince CL Whiting KE Wang T Lékó AH Farinelli LA Cooper D. Role of aldosterone and mineralocorticoid receptor in addiction: a scoping review. Neurosci Biobehav Rev. (2023) 154:105427. 10.1016/j.neubiorev.2023.105427 37858908 PMC10865927

[30] Monahan KD Leuenberger UA Ray CA. Aldosterone impairs baroreflex sensitivity in healthy adults. Am J Physiol Heart Circ Physiol. (2007) 292(1):H190–7. 10.1152/ajpheart.00622.2006 16920805

[31] Wang Y Li CX Lin YN Zhang LY Li SQ Zhang L. The role of aldosterone in OSA and OSA-related hypertension. Front Endocrinol (Lausanne). (2022) 12:801689. 10.3389/fendo.2021.801689 35095768 PMC8791261

[32] Gaddam K Corros C Pimenta E Ahmed M Denney T Aban I. Rapid reversal of left ventricular hypertrophy in resistant hypertension and hyperaldosteronism. Hypertension. (2010) 55:1137–42. 10.1161/HYPERTENSIONAHA.109.141531 20351345 PMC2864599

[33] Lin C-H Lin C-H Chung M-C Hung C-S Tseng F-Y Er LK. Aldosterone-to-renin ratio as screening tool for primary aldosteronism. J Formos Med Assoc. (2024) 123(Suppl 2):S98–S103. 10.1016/j.jfma.2023.04.019 37173226

[34] Yozamp N Hundemer GL Moussa M Underhill J Fudim T Sacks B. Intraindividual variability of aldosterone concentrationsin primary aldosteronism. Hypertension. (2021) 77:891–9. 10.1161/HYPERTENSIONAHA.120.16429 33280409 PMC7878320

[35] Kecaj I Nelaj E Gjermeni I Xhixhabesi K Refatllari I. Primary hyperaldosteronism: an underdiagnosed syndrome. Arch Balk Med Union. (2024) 59(4):409–14. 10.31688/ABMU.2024.59.4.11

[36] Vasan RS Evans JC Larson MG Wilson PWF Meigs JB Rifai N. Serum aldosterone and incidence of hypertension in nonhypertensive persons. N Engl J Med. (2004) 351(1):33–41. 10.1056/NEJMoa033263 15229305

[37] Sartori M Calo L Mascagna V Realdi A Macchini L Ciccariello L. Aldosterone and refractory hypertension: a prospective cohort study. Am J Hypertens. (2006) 19(4):373–9. 10.1016/j.amjhyper.2005.06.031 16580572

[38] Lamba R. Redefining primary hyperaldosteronism as syndrome of inappropriate aldosterone secretion (SIALDS)”: a common but unrecognized cause of hypertension. J Clin Hypertens (Greenwich). (2023) 25(12):1045–52. 10.1111/jch.14740 37877173 PMC10710549

[39] Hung A Ahmed S Gupta A Davis A Kline GA Leung AA. Performance of the aldosterone-to-renin ratio as a screening test for primary aldosteronism. J Clin Endocrinol Metab. (2021) 106:2423–35. 10.1210/clinem/dgab348 34008000

[40] Marcelli M Bi C Funder JW McPhaul MJ. Comparing ARR versus suppressed PRA as screening tests for primary aldosteronism. Hypertension. (2024) 81(10):2072–81. 10.1161/HYPERTENSIONAHA.124.22884 39041222

[41] Brown JM Siddiqui M Calhoun DA Carey RM Hopkins PN Williams GH. Unrecognized prevalence of primary aldosteronism. Ann Intern Med. (2020) 173:10–20. 10.7326/M20-0065 32449886 PMC7459427

[42] Galuppo P Bauersachs J. Mineralocorticoid receptor activation in myocardial infarction and failure: recent advances. Eur J Clin Invest. (2012) 42(10):1112–20. 10.1111/j.1365-2362.2012.02676.x 22536780

[43] Wong KYK Wong SYS McSwiggan S Ogston SA Sze KYS MacWalter RS. Myocardial fibrosis and QTc reduction following spironolactone or amiloride in stroke survivors: a randomised placebo-controlled cross-over trial. Int J Cardiol. (2013) 168(6):5229–33. 10.1016/j.ijcard.2013.08.027 23993727

[44] Wolf J Narkiewicz K. Managing cardiovascular disease and sleep apnea with pharmacotherapy. Expert Opin Pharmacother. (2018) 19(9):961–9. 10.1080/14656566.2018.1476489 29792524

[45] Chen Z-W Pan C-T Liao C-W Tsai C-H Chang Y-Y Chang C-C. Implication of MR activity in posttreatment arterial stiffness reversal in patients with primary aldosteronism. J Clin Endocrinol Metab. (2022) 108:624–32. 10.1210/clinem/dgac649 36333943

[46] Sechi LA Colussi G Di Fabio A Catena C. Cardiovascular and renal damage in primary aldosteronism: outcomes after treatment. Am J Hypertens. (2010) 23:1253–60. 10.1038/ajh.2010.169 20706195

[47] Pan CT Tsai CH Chen ZW Chang YY Wu VC Hung CS. Atrial fibrillation in primary aldosteronism. Horm Metab Res. (2020) 52:357–65. 10.1055/a-1141-5989 32289838

[48] Williams RS Delemos JA Dimas V Reisch J Hill JA Naseem RH. Effect of spironolactone on atrial fibrillation in structural heart disease. Clin Cardiol. (2011) 34(7):415–9. 10.1002/clc.20914 21674535 PMC3617486

[49] Swedberg K Zannad F McMurray JJV Krum H Van Veldhuisen DJ Shi H. Eplerenone and atrial fibrillation in mild systolic heart failure. J Am Coll Cardiol. (2012) 59(18):1598–603. 10.1016/j.jacc.2011.11.063 22538330

[50] Pitt B Pfeffer MA Assmann SF Boineau R Anand IS Claggett B. Spironolactone for heart failure with preserved ejection fraction. N Engl J Med. (2014) 370:1383–92. 10.1056/NEJMoa1313731 24716680

[51] Zannad F McMurray JJV Krum H Van Veldhuisen DJ Swedberg K Shi H. Eplerenone in patients with systolic heart failure and mild symptoms. N Engl J Med. (2011) 364:11–21. 10.1056/NEJMoa1009492 21073363

[52] Pitt B Zannad F Remme WJ Cody R Castaigne A Perez A. The effect of spironolactone on morbidity and mortality in patients with severe heart failure. Randomized aldactone evaluation study investigators. N Engl J Med. (1999) 341(10):709–17. 10.1056/NEJM199909023411001 10471456

[53] Rossi GP Cesari M Cuspidi C Maiolino G Cicala MV Bisogni V. Long-Term control of arterial hypertension and regression of left ventricular hypertrophy with treatment of primary aldosteronism. Hypertension. (2013) 62:62–9. 10.1161/HYPERTENSIONAHA.113.01316 23648698

[54] Saiki A Otsuki M Tamada D Kitamura T Mukai K Yamamoto K. Increased dosage of MRA improves BP and urinary albumin excretion in primary aldosteronism with suppressed plasma renin. J Endocr Soc. (2022) 6(1):bvab174. 10.1210/jendso/bvab174 34909517 PMC8664755

