# Supplementary material for: Subclinical primary aldosteronism and major adverse cardiovascular events: evidence for a continuum of renin-independent aldosterone excess and a proposal for early detection
Source: Front Cardiovasc Med. 2026 Jun 23;13:1826678. doi: 10.3389/fcvm.2026.1826678 (PMC13338875; doi:10.3389/fcvm.2026.1826678)
Supplement: Supplementary Table S1 — Newcastle-Ottawa Scale quality assessment of included studies. [file Supplementaryfile1.docx]

Supplementary Material

# Supplementary Figures and Tables

## Supplementary Figures

**Supplementary Figure 1:** PRISMA flow diagram

**
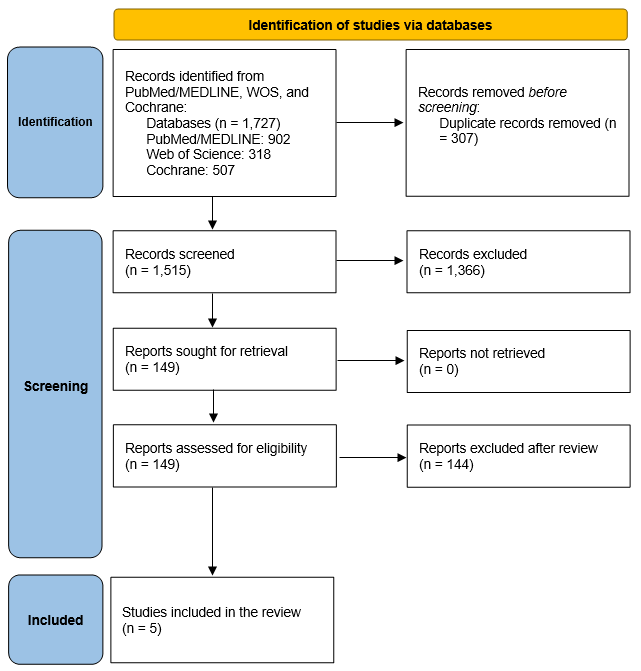
**

## 1.2 Supplementary Tables

**Supplementary Table 1.** Newcastle-Ottawa Scale quality assessment of included studies.

| **NOS Criterion** | **Vasan 2004** | **Markou 2013** | **Brown 2017** | **Hundemer 2024** | **Goupil 2025** |
| --- | --- | --- | --- | --- | --- |
| **Selection domain (max 4 points)** | | | | | |
| S1. Representativeness of exposed cohort Community/population-based only; not clinic/hospital/volunteer | **Met —** Framingham community cohort; random sample | **Not met/NR —** Clinic-based thyroid/bone referral; not community-representative | **Met —** MESA multi-center community cohort; population-representative | **Met —** CARTaGENE randomly sampled population cohort | **Met —** CARTaGENE randomly sampled population cohort |
| S2. Selection of non-exposed cohort From same community as exposed | **Met —** Same Framingham cohort; non-exposed quartile from same source | **Met —** Controls from same clinic pool | **Met —** Non-suppressed PRA groups from same MESA cohort | **Met —** Continuous exposure; lower ARR group from same cohort | **Met —** Continuous exposure; reference group from same cohort |
| S3. Ascertainment of exposure Validated biochemical assay with structured blood draw | **Met —** Validated radioimmunoassay; seated structured blood draw | **Met —** Validated RIA for ARR and aldosterone; structured protocol | **Met —** Validated radioimmunoassay; structured draws per protocol | **Met —** Validated chemiluminescent immunoassay; blood collected on ice | **Met —** Validated chemiluminescent immunoassay; standardized protocol |
| S4. Outcome not present at start Outcome explicitly absent at baseline | **Met —** Hypertension explicitly absent at baseline; normotensive cohort | **Met —** Normotension confirmed at baseline by definition of entry criteria | **Met —** Normotensive and antihypertensive-free at entry; explicitly stated | **Met —** Normotensive subgroup analysis; HTN-free at baseline stated | **Not met/NR —** 3% of participants had prior CVD at baseline; outcome not absent for all |
| **Comparability domain (max 2 points)** | | | | | |
| C1. Controlled for age, sex, AND blood pressure All three required simultaneously | **Met —** Adjusted for age, sex, and baseline BP category simultaneously | **Not met/NR —** Age and sex not included in final multivariable regression model | **Met —** Adjusted for age, sex, and systolic BP in primary model | **Met —** Adjusted for age, sex, and brachial systolic BP | **Met —** Adjusted for age, sex, and mean arterial pressure |
| C2. Additional confounders At least two of: BMI, DM, smoking, medications, eGFR, potassium | **Met —** Adjusted for BMI, diabetes, smoking, weight gain, heart rate | **Met —** Adjusted for BMI and serum potassium | **Met —** Adjusted for BMI, smoking, LDL, fasting glucose, eGFR | **Met —** Adjusted for BMI, eGFR, serum Na/K, diabetes, statin use, antihypertensives | **Met —** Adjusted for serum potassium, dietary sodium, RAAS inhibitors, eGFR |
| **Outcome domain (max 3 points)** | | | | | |
| O1. Assessment of outcome Blind assessment, record linkage, or structured clinical examination | **Met —** Structured physician examination with mercury sphygmomanometry | **Met —** Validated biochemical confirmatory testing (FDST); 5-year clinical follow-up | **Met —** Automated BP measurement and medication inventory; standardized | **Met —** Standardized BP measurement and core-lab cardiac MRI examination | **Met —** Validated record linkage to RAMQ provincial health database |
| O2. Follow-up length Min 4 years for HTN; min 5 years for MACE; not awarded for cross-sectional structural outcomes | **Met —** 4-year follow-up for incident hypertension; meets minimum | **Met —** 5-year follow-up for incident hypertension; meets minimum | **Met —** 10-year follow-up for incident hypertension; exceeds minimum | **Met —** 5 to 7-year follow-up for incident HTN; MRI structural outcomes prospective | **Met —** Median 10.8-year follow-up for hard MACE outcomes; exceeds minimum |
| O3. Adequacy of follow-up Loss to follow-up <20% or explicitly described with handling method | **Met —** 115/3375 loss to follow-up; explicitly reported; well under 20% | **Met —** Approximately 10% attrition; reported and comparable between groups | **Not met/NR —** Attrition rate for 850-person normotensive subset not explicitly reported | **Not met/NR —** Specific attrition from starting pool for this sub-study not described | **Met —** Only 2% loss to follow-up; explicitly reported |
| **TOTAL NOS SCORE** | **9/9** | **7/9ᵃ** | **8/9** | **8/9** | **8/9ᵇ** |

Each item was assessed using information reported in the published article. Criteria that were not clearly reported were classified as not met or not reported. The total score is presented out of 9 points.

Markou et al. 2013 scored 7/9. Points were not awarded for representativeness because the cohort was clinic-based, and for comparability because age and sex were not included in the final multivariable model. The small PA-positive subgroup should be considered when interpreting the effect estimate.

Goupil et al. 2025 scored 8/9. The baseline absence of outcome was not met because 3% of participants had prior cardiovascular disease. The proposed biochemical thresholds were derived post hoc and therefore require prospective validation before clinical use.

Abbreviations: NOS, Newcastle-Ottawa Scale; PRA, plasma renin activity; ARR, aldosterone-to-renin ratio; BP, blood pressure; BMI, body mass index; DM, diabetes mellitus; eGFR, estimated glomerular filtration rate; FDST, fludrocortisone-dexamethasone suppression test; MESA, Multi-Ethnic Study of Atherosclerosis; MACE, major adverse cardiovascular events; RAMQ, Regie de l’assurance maladie du Quebec; HTN, hypertension; CVD, cardiovascular disease; RIA, radioimmunoassay; MRI, magnetic resonance imaging; RAAS, renin-angiotensin-aldosterone system; PA, primary aldosteronism.
